# Effect of Limit-Fed Diets With Different Forage to Concentrate Ratios on Fecal Bacterial and Archaeal Community Composition in Holstein Heifers

**DOI:** 10.3389/fmicb.2018.00976

**Published:** 2018-05-15

**Authors:** Jun Zhang, Haitao Shi, Yajing Wang, Zhijun Cao, Hongjian Yang, Shengli Li

**Affiliations:** ^1^State Key Laboratory of Animal Nutrition, Beijing Engineering Technology Research Center of Raw Milk Quality and Safety Control, College of Animal Science and Technology, China Agricultural University, Beijing, China; ^2^Department of Animal and Poultry Science, University of Saskatchewan, Saskatoon, SK, Canada

**Keywords:** bacteria, archaea, heifer, forage to concentrate ratio, feces

## Abstract

Limit-feeding of a high concentrate diet has been proposed as an effective method for improving feed efficiency and reducing total manure output of dairy heifers; meanwhile the effects of this method on hindgut microbiota are still unclear. This study investigated the effects of a wide range of dietary forage:concentrate ratios (F:C) on the fecal composition of bacteria and archaea in heifers using next-generation sequencing. Four diets with different F:C (80:20, 60:40, 40:60, and 20:80) were limit-fed to 24 Holstein heifers, and the fecal fermentation parameters and bacterial and archaeal communities were investigated. With increasing dietary concentrate levels, the fecal dry matter output, neutral detergent fiber (NDF) content, and proportion of acetate decreased linearly (*P* < 0.01), while the fecal starch content and proportions of propionate, butyrate, and total branched-chain volatile fatty acids (TBCVFAs) were increased (*P* ≤ 0.05). An increased concentrate level linearly increased (*P* = 0.02) the relative abundance of *Proteobacteria*, and linearly decreased (*P* = 0.02) the relative abundance of *Bacteroidetes* in feces. At the genus level, the relative abundance of unclassified Ruminococcaceae and *Paludibacter* which may have the potential to degrade forage decreased linearly (*q* ≤ 0.02) with increasing dietary concentrate levels, while the relative abundance of *Roseburia* and *Succinivibrio* which may be non-fibrous carbohydrate degrading bacteria increased linearly (*q* ≤ 0.05). Some core microbiota operational taxonomic units (OTUs) also showed significant association with fecal VFAs, NDF, and/or acid detergent fiber (ADF) content. Meanwhile, the relative abundance of most detected taxa in archaea were similar across different F:C, and only *Methanosphaera* showed a linear decrease (*P* = 0.01) in high concentrate diets. Our study provides a better understanding of fecal fermentation parameters and microbiota under a wide range of dietary F:C. These findings support the potential for microbial manipulation by diet, which could enhance feed digestibility and relieve environmental problems associated with heifer rearing.

## Introduction

Ruminants are uniquely able to convert large quantities of plant fiber into high quality products, such as meat and milk, for human consumption. With the current rapidly exploding human population, there is a great need to increase ruminant productivity and feed conversion efficiency (Thornton, 2010). Recently, the limit-feeding of high concentrate diets has been proposed as an effective method for improving feed efficiency in heifers, and has been explored by many researchers (Suarez-Mena et al., 2015; Lascano et al., 2016; Zanton and Heinrichs, 2016). Furthermore, when heifers are raised under a limit-fed diet of enhanced energy density, intakes of feed and forage are reduced, as is fecal or total manure output (Moody et al., 2007; Zanton and Heinrichs, 2009); this is an environmentally friendly dairy farming strategy. Previous studies report that wet fecal output for high concentrate fed heifers represents 64–66% of the amounts excreted from low concentrate fed heifers (Lascano et al., 2016; Zanton and Heinrichs, 2016). However, the effects of limit-feeding different forage to concentrate ratios (F:C) on hindgut microbiota communities are still unclear.

Microbiota of the gastrointestinal tract (GIT) have an important role in animal health, welfare, and productivity, and also in food safety, pathogen shedding, and the detection of fecal microbial contamination in water or lands by microbial source tracking (Lee et al., 2011; Shanks et al., 2011; Rice et al., 2012). Previous studies report that diets and/or feeding strategy mainly determine cattle fecal bacterial community structure (Callaway et al., 2010; Shanks et al., 2011; Kim et al., 2014). However, the GIT microbiome is quite complicated, and other microbes like archaea, which are solely responsible for methane production, also live in the ruminant gut (Kumar et al., 2015; Zhang et al., 2017). Methane (CH_4_) emitted from ruminants accounts for 4–12% of gross energy loss, and also causes environmental problems due to its remarkable global warming potential (Johnson and Johnson, 1995; Kumar et al., 2015). Manure CH_4_ from ruminant livestock may contribute up to 2% of global CH_4_ emissions; moreover, manure CH_4_ emissions are a larger proportion of total farm CH_4_ emissions in intensively managed dairy operations with manure storage systems (Knapp et al., 2014). Therefore, a better understanding of fecal bacterial and archaeal communities under a wide range of F:C diets could be important in improving feed utilization, manipulating digestion, further increasing livestock growth performance, and even reducing related environmental problems.

Nowadays, high-through sequencing techniques have been successfully used to reveal changes and function of the microbial community of the GIT (Mao et al., 2015; Ye et al., 2016; Zhang et al., 2017). Therefore, the primary objective of this study was to evaluate the effects of a wide range of dietary F:C (four levels) on the fecal composition of bacteria and archaea in heifers using next-generation sequencing.

## Materials and methods

### Animals and diets

Twenty-four half-sib Holstein heifers (8–10 months old and 263 ± 30 kg body weight) with similar initial body condition (2.73 ± 0.03 using a five-point system body condition score) were assigned into one of four F:C treatments: 80:20, 60:40, 40:60, and 20:80, on a dry matter (DM) basis; the groups were named C20, C40, C60, and C80, respectively (Table S1). Corn silage was used as the sole forage resource, as reported in our previous study (Zhang et al., 2018). Before transfer to treatment diets, all heifers were fed an adaptation diet containing 50% forage and 50% concentrate (DM basis; Table S1) for 4 weeks. Diets were designed according to the nutrient requirements for dairy heifers (NRC, 2001). Diet quantity provided to the high concentrate groups was restricted to give a similar intake of metabolizable energy (ME) to the low concentrate groups. Additionally, diets were formulated to maintain the same crude protein (CP):ME ratios to minimize potential effects due to differences in CP intake across diets. Heifers were individually fed a total mixed ration (TMR) twice daily at 12 h intervals (07:00 and 19:00 h) and weighed weekly 2 h prior to the morning feeding (0500 h) for the adjustment of feed offered. Water was available ad libitum.

This study was carried out in Sanyuan Dairy Group, Beijing, China, and in accordance with the recommendations of Instructive Notions with Respect to Caring for Experimental Animals, Ministry of Science and Technology of China. The protocol was approved by the Ethical Committee of the College of Animal Science and Technology (Project number 31402099) of China Agricultural University.

### Sampling and measurements

To obtain representative samples, fecal samples (about 200 g/sampling) were collected from the rectum of heifers during days 20–23 of the experimental period (except for one sick heifer from the C60 group) using sterile gloves. On days 20 and 22, collection was at 08:00, 14:00, and 20:00 h, while on days 21 and 23, collection was at 02:00, 05:00 h, 11:00, 17:00, and 23:00 h to achieve a combined 2-day sample taken at 3 h intervals. After each sampling time point, half the sample was immediately stored in liquid nitrogen and the remainder was stored at −20°C. At the end of sampling, equal amounts of the liquid nitrogen samples from each time point and from the same heifer were mixed to homogeneity using a Slapping Sterile Homogenizer (Shanghai Hannuo Ltd., Shanghai China), and subsamples of ~1 g were frozen at −80°C in the laboratory for further genomic DNA extraction. In addition, equal amounts of the −20°C samples from each time point and from the same heifer were mixed to homogeneity, and subsamples of ~2 g were dissolved with 2 mL of water for the detection of volatile fatty acids (VFAs) (Mao et al., 2012). The remaining fecal samples were analyzed for DM (AOAC, 2000; method 930.15), organic matter (OM) (Van Soest et al., 1991), CP (AOAC, 2000; method 976.05), neutral detergent fiber (NDF; with a heat stable amylase and expressed inclusive of residual ash), and acid detergent fiber (ADF) (Van Soest et al., 1991). Fecal starch content was analyzed using a total starch assay kit (Megazyme, Bray, Ireland) based on AOAC method 996.11 (AOAC, 2000).

### Microbial DNA extraction, PCR amplification, and sequencing

Genomic DNA was extracted from the fecal samples using a FastDNA SPIN Kit (MP Biomedicals, Solon, USA) following the manufacturer's protocol. The extracted DNA was subjected to PCR amplification in triplicate using a KAPA HiFi Hotstart ReadyMix PCR kit (Life Technology, Carlsbad, USA). For bacteria, the V3–V4 region of the bacteria 16S rRNA gene was amplified using primers F341 (5′- ACTCCTACGGGRSGCAGCAG−3′) and R806 (5′- GGACTACVVGGGTATCTAATC−3′) (Sun et al., 2017). For archaea, the V3–V4 region of the archaea 16S rRNA gene was amplified using primers F341 (5′- CTACGGGGYGCASCAG′) (Wei et al., 2013) and R806 (5′- GGACTACVVGGGTATCTAATC−3′). Barcodes, an eight-base sequence unique to each sample, were added to each primer for sample identification. Amplification was performed as follows: initial denaturing step of 95°C for 3 min, followed by 25 cycles of 95°C for 30 s, annealing (60°C for bacteria/55°C for archaea, 20 s), elongation (72°C, 40 s), and a final extension step of 72°C for 10 min. Amplicons were extracted from 2% agarose gels and purified using an AxyPrep DNA Gel Extraction Kit (Axygen Bioscience, Union City, CA, USA) following the manufacturer's instructions and quantified using a NanoDrop 2000 (Thermo, Mass, USA). Illumina TruSeq PE Cluster and SBS kits (Illumina, San Diego, USA) were used to perform cluster generation, template hybridization, isothermal amplification, linearization, blocking, denaturization, and hybridization of the sequencing primers according to the manufacturer's instruction. Paired-end sequencing (2 × 250 bp) was performed to sequence all libraries on an Illumina HiSeq2500 PE250 platform (Illumina, USA) according to standard protocols (Caporaso et al., 2012).

### Sequence data processing

The sequences obtained from the HiSeq platform were processed through an open-source software pipeline, Quantitative Insights into Microbial Ecology (QIIME) version 1.8.0-dev (Caporaso et al., 2010b), with criteria similar to a previous report (Zhang et al., 2017). Briefly, (1) the reads with an average quality score of no less than 25 and with a length of 220–250 nt were retained; (2) reads that had more than three N bases, two nucleotide mismatches in primer matching, and ambiguous characters were discarded; (3) reads that could not be assembled were discarded. Sequences were binned into operational taxonomic units (OTUs) based on 97% identity using USEARCH after removing the singletons, and chimeric sequences were identified and removed by UCHIM (Edgar, 2013). For bacterial sequencing analysis, three samples with quite low numbers of OTUs were excluded. Thus, 20 fecal samples were finally used to assess the bacterial community, with five samples from each group. The most abundant sequence within each OTU from specific libraries (libraries constructed for bacteria and archaea) was designated as the “representative sequence” and then aligned against the SILVA bacterial database (SILVA version 123) (Pruesse et al., 2007) and SILVA archaea database (SILVA version 123) using PYNAST (Caporaso et al., 2010a), respectively, with default parameters set by QIIME. Community richness and diversity, such as Good's coverages, Observed species, PD whole tree, Chao1, Shannon, and Simpson indices, which are used to illustrate significant differences among samples, were assessed by the program MOTHUR v.1.35.0 (Schloss et al., 2009). Beta diversity was measured according to unweighted UniFrac distances which were calculated by QIIME, and displayed using Principal Coordinate Analysis (PCoA). The significance of grouping in the PCoA plot was tested by analysis of similarity (ANOSIM) in QIIME with 999 permutations (R Core Team, 2014).

After assembling and filtering, all the raw sequences (bacteria and archaea) were submitted to the NCBI Sequence Read Archive (SRA; http://www.ncbi.nlm.nih.gov/Traces/sra/), under accession number SRP117671.

### Quantitative real-time PCR analysis

Marker loci gene copies for total bacteria, total Methanogens, the genus *Prevotella, Fibrobacter succinogenes, Ruminococcus albus*, and *Ruminococcus flavefaciens* were enumerated by quantitative PCR on a QuantStudio 6 Flex Real-Time PCR System (Thermo Scientific, Rockford, IL, USA) using SYBR Green as the fluorescent dye. The primers and reaction conditions were selected on the basis of a careful review of published literature (Table S2). The standard curves were generated from diluting plasmid DNA (10-fold serial dilution) that contained the cloned marker loci. Each reaction contained 5 μL of 2 × SYBR Premix Ex Taq II (Tli RNaseH Plus) (TaKaRa Bio Inc., Shiga, Japan), 1 μL of each primer (10 μM), 0.2 μL of Rox (Takara Bio), 80 ng of microbial DNA, and 2.3 μL of deionized water. Thermal cycling was performed at 95°C for 30 s, followed by 40 cycles of 95°C for 15 s, annealing for 30–60 s (annealing temperatures and times are shown in Table S2), and collection of fluorescent signals. All samples were prepared from the heifers and each sample was assayed in triplicate. All obtained standard curves met the required standards of efficiency (*R*^2^ > 0.99, 110% > E > 90%). Results were expressed as the log_10_ transfer of numbers of marker loci gene copies per gram of feces (wet weight).

### Correlations between the fecal microbiota OTUs and nutrient content and VFAs concentrations

OTUs that appeared in all the samples with an abundance > 0.001 were considered as core OTUs (Table S3). Fecal fermentation parameters, nutrient contents, and core microbiota OTUs which can be identified at family and/or genus levels were used for interactive analysis in R (R Core Team, 2014). Spearman's rank correlations and *q*-values were calculated using the Psych packages [http://cran.r-project.org/web/packages/psych; author, W. Revelle; published date, 2016; version, 1.6.9; FDR correct (*q*-value) was embedded in the package] and visualized using the corrplot package (https://cran.r-project.org/web/packages/corrplot/; author, Taiyun Wei; published date, 2017; version, 0.84) in R. Correlations had an absolute Spearman's correlation > 0.50 with a *q*-value under 0.05.

### Statistical analysis

Statistical analyses were performed using R software packages. The dietary effects were tested using an estimability package (https://cran.r-project.org/package=estimability; author: R. V. Lenth; published date: 2015; version: 1.1-1) in R. The lm function of estimability package in R was used to evaluate the linear, quadratic, and cubic effects of the dietary concentrate levels on the variables. Standard errors of the mean were reported. Significance was declared at *P* ≤ 0.05, and trend was reported at 0.05 < *P* < 0.10. Furthermore, all *P-*values from the multiple comparison analyses of the bacterial community (genus level) were adjusted by the FDR using the BonEV package (https://cran.r-project.org/package=BonEV; author, D. Li; published date, 2016; version, 1.0) with p.adjust in R. FDR-corrected *P-*values ≤ 0.05 (*q* ≤ 0.05) were considered a significant difference.

## Results

### Fecal nutrient and VFAs content

Fecal DM output and OM, NDF, and ADF content decreased linearly (*P* < 0.01) with increasing amounts of concentrate in the diets, while CP and starch content increased linearly (*P* ≤ 0.05; Table 1). The proportion of acetate and acetate:propionate ratios in feces decreased linearly (*P* ≤ 0.01) with increasing dietary concentrate levels, while the proportion of butyrate was increased linearly (*P* < 0.01). The proportion of propionate increased quadratically (*P* = 0.03) with increasing dietary concentrate levels with the C60 group having the highest value. The proportions of isobutyrate, isovalerate, and total branched-chain volatile fatty acids (TBCVFA) increased quadratically (*P* = 0.01) with the C40 group having the lowest values.

**Table 1:** Fecal nutrient contents and VFAs concentrations.

**Items^a^**	**Treatments**				**SEM^b^**	***P*****-value**		
	**C20**	**C40**	**C60**	**C80**		**Linear**	**Quadratic**	**Cubic**
Fecal DM output, kg/day^c^	1.20	1.02	0.91	0.85	0.034	< 0.01	0.16	0.80
OM, % of DM	81.03	80.04	79.82	75.56	0.639	< 0.01	0.04	0.19
CP, % of DM	14.42	17.14	18.87	19.99	0.547	< 0.01	0.19	0.89
NDF, % of DM	61.69	58.98	59.38	50.94	1.180	< 0.01	0.08	0.09
ADF, % of DM	36.89	34.63	32.71	27.80	0.800	< 0.01	0.11	0.35
Starch, % of DM	0.60	1.38	1.46	2.69	0.322	0.05	0.75	0.57
TVFA, mM	34.67	41.36	46.80	37.95	3.850	0.37	0.05	0.44
**VFAs, MOLAR % OF TVFA**								
Acetate	68.35	66.80	65.07	61.95	1.015	< 0.01	0.43	0.78
Propionate	17.10	18.16	18.46	17.57	0.419	0.36	0.03	0.82
Butyrate	8.29	9.84	10.86	13.11	0.693	< 0.01	0.61	0.56
Valerate	1.65	1.51	1.58	1.70	0.092	0.54	0.16	0.68
Isobutyrate	2.73	2.11	2.28	3.01	0.246	0.34	0.01	0.83
Isovalerate	1.89	1.57	1.75	2.65	0.214	0.02	0.01	0.79
TBCVFA	4.62	3.68	4.03	5.67	0.281	0.09	0.01	0.99
A:P	4.01	3.70	3.53	3.54	0.122	0.01	0.20	0.90

a *DM, dry matter; OM, organic matter; CP, crude protein; NDF, neutral detergent fiber; ADF, acid detergent fiber; TVFA, total volatile fatty acid; TBCVFA, total branch-chain volatile fatty acids; A:P, acetate:propionate*.

b *SEM, standard error of the mean*.

c *Estimate by DM intake × (1- apparent DM digestibility); the values of DM intake and apparent DM digestibility were shown in our previous study (Zhang et al., 2018)*.

*C20, diet contained 20% of concentrate; C40, diet contained 40% of concentrate; C60, diet contained 60% of concentrate; C80, diet contained 80% of concentrate*.

### Sequence and general bacterial community composition

A total of 687,332 quality-checked sequences were obtained from all the 20 samples, and 30,518–38,910 sequences were returned for each sample. After OTU picking and chimera checking, a total of 1,091 OTUs were calculated for all the samples at 3% dissimilarity. The number of OTUs for the C20, C40, C60, and C80 groups were 752, 720, 710, and 715, respectively; furthermore, 801 OTUs were found in all four treatments (Figure S1A). Good's coverage for each sample was more than 0.99.

**Figure 1:**
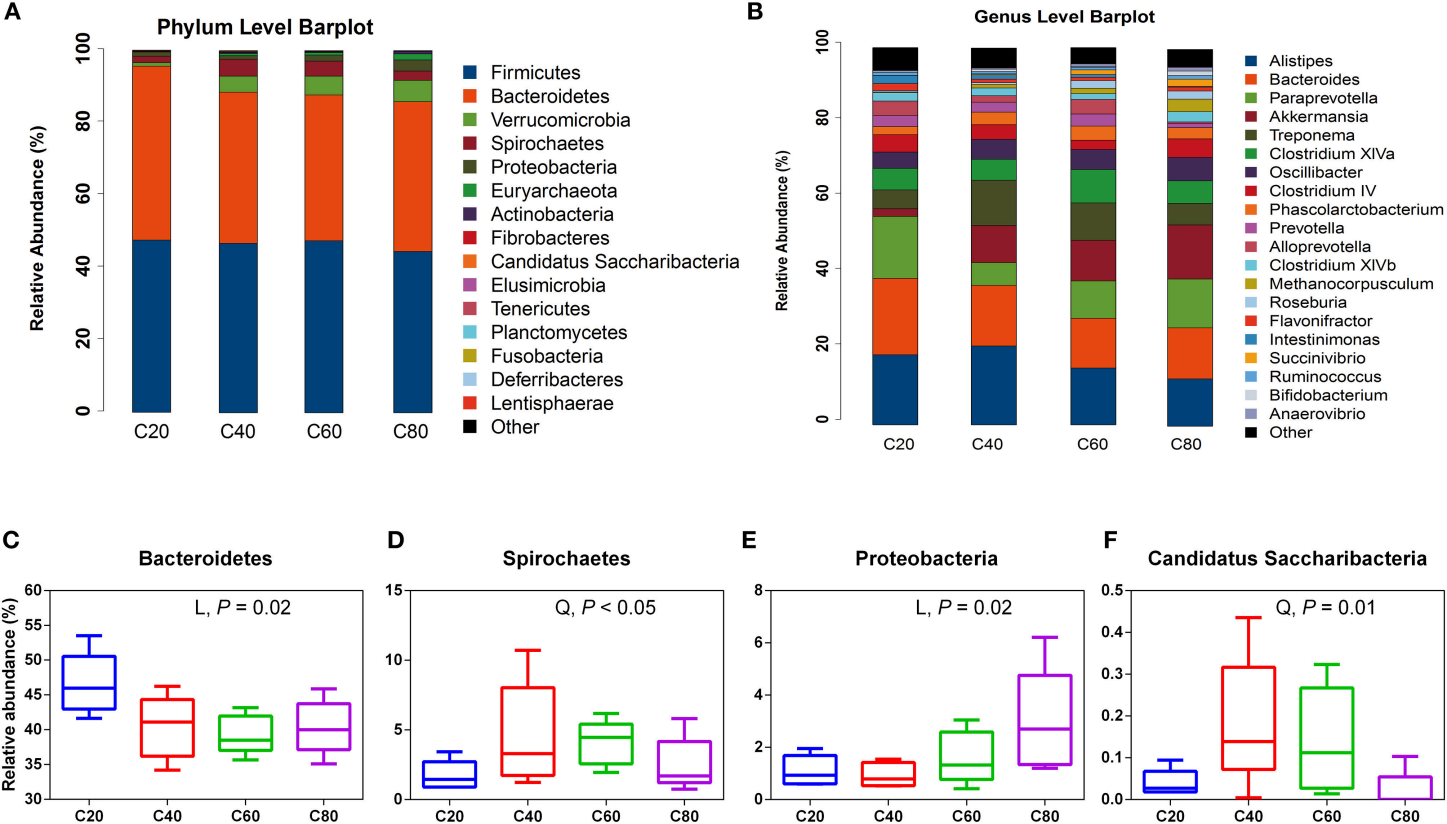
Fecal bacterial phyla and genera in four treatments. **(A)** The bacterial taxonomic composition of fecal samples from the four treatments at the phylum level. **(B)** The bacterial taxonomic composition of fecal samples from the four treatments at the genus level (top 20, according to relative abundance). The significantly changed bacterial phyla *Bacteroidetes* **(C)**, *Spirochaetes* **(D)**, *Proteobacteria* **(E)**, and Candidatus *Saccharibacteria* **(F)** among treatments. The small box plots show the 25, 50, and 75th percentiles, with whiskers showing the extremes of the data. L, changed linearly; Q, changed quadratically. C20, diet containing 20% of concentrate; C40, diet containing 40% of concentrate; C60, diet containing 60% of concentrate; C80, diet containing 80% of concentrate.

A total of 14 phyla were detected via taxonomic analyses. The five most abundant phyla were *Firmicutes, Bacteroidetes, Verrucomicrobia, Spirochaetes*, and *Proteobacteria*, representing 45.34, 41.67, 4.02, 3.22, and 1.66% of total sequences, respectively (Figure 1A). At the genus level, a total of 92 bacterial genera were detected, and 33 genera were common in all samples; these were identified as the core bacterial microbiome (Figure S2A). The seven most abundant genera were unclassified Ruminococcaceae, *Alistipes, Bacteroides, Paraprevotella*, unclassified Porphyromonadaceae, unclassified Lachnospiraceae, and *Akkermansia*, representing 22.53, 6.54, 6.00, 4.63, 4.55, 3.74, and 3.61% of total sequences, respectively (Figure 1B).

### Effects of increasing dietary concentrate levels on fecal bacterial communities

After rarefaction, 22,000 sequences per sample were used for diversity analysis. Alpha bacterial diversity is presented in Table 2. Similar values for Chao1, Observed species, and PD whole tree indices were observed among the four groups (*P* > 0.05). The diversity indices (Shannon and Simpson indices) linearly decreased (*P* < 0.01) with increasing concentrate levels in diets.

**Table 2:** Alpha diversity index of fecal bacteria and archaea among all treatments.

**Microbiota**	**Indices**	**Treatments**				**SEM^a^**	***P*****-value**		
		**C20**	**C40**	**C60**	**C80**		**Linear**	**Quadratic**	**Cubic**
Bacteria	Observed species	728.80	702.40	698.80	689.80	9.676	0.22	0.68	0.77
	PD whole tree	40.69	39.61	39.78	40.89	0.370	0.86	0.28	0.95
	Chao1	801.51	810.84	816.56	799.53	4.619	1.00	0.60	0.87
	Shannon	7.69	7.46	7.39	6.94	0.180	< 0.01	0.39	0.36
	Simpson	0.99	0.99	0.99	0.97	0.004	0.01	0.07	0.28
Archaea	Observed species	406.67	363.17	448.20	368.50	22.782	0.82	0.55	0.04
	PD whole tree	31.38	29.05	33.53	30.00	1.123	0.96	0.69	0.04
	Chao1	497.82	445.60	530.51	477.10	20.600	0.89	0.99	0.13
	Shannon	2.97	2.22	3.26	2.02	0.342	0.37	0.59	0.06
	Simpson	0.69	0.50	0.75	0.47	0.080	0.29	0.62	0.02

a *SEM, standard error of the mean*.

*C20, diet contained 20% of concentrate; C40, diet contained 40% of concentrate; C60, diet contained 60% of concentrate; C80, diet contained 80% of concentrate*.

**Figure 2:**
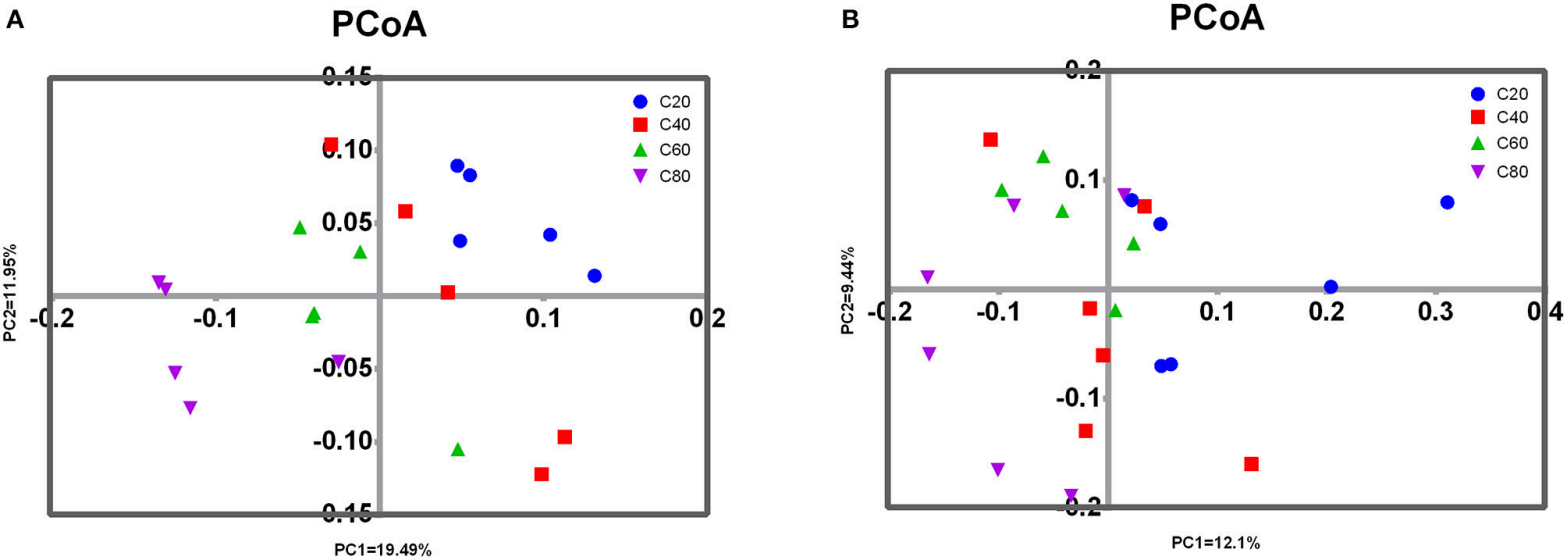
Principal coordinated analysis (PCoA) of fecal microbial communities. Unweighted PCoA by fecal bacteria **(A)** and archaea **(B)** microbiota. C20, diet containing 20% of concentrate; C40, diet containing 40% of concentrate; C60, diet containing 60% of concentrate; C80, diet containing 80% of concentrate.

PCoA analysis of overall diversity based on unweighted UniFrac metrics was performed to compare the four treatments (Figure 2A). ANOSIM showed significant differences in bacterial community composition between treatments C20 and C60 (*R* = 0.476, *P* < 0.01), between treatments C20 and C80 (*R* = 0.836, *P* = 0.01), and between treatments C40 and C80 (*R* = 0.572, *P* = 0.02). A tendency of difference was found between treatments C20 and C40 (*R* = 0.172, *P* = 0.08) and between treatments C60 and C80 (*R* = 0.268, *P* = 0.08). No significant difference was found between treatments C40 and C60 (*R* = 0.140, *P* = 0.16).

At the phylum level, the relative abundance of *Proteobacteria* increased linearly (*P* = 0.02) with increasing dietary concentrate levels, while the relative abundance of *Bacteroidetes* decreased linearly (*P* = 0.02; Table 3, Figures 1C,E). The relative abundances of Candidatus *Saccharibacteria* and *Spirochaetes* quadratically decreased (*P* < 0.05; Figures 1D,F) with increasing dietary concentrate levels, with the C40 group having the highest values. At the genus level, the relative abundances of *Roseburia, Succinivibrio*, and *Clostridium sensu strict* increased linearly (*q* ≤ 0.05) with increasing dietary concentrate levels, while the relative abundances of unclassified Ruminococcaceae, *Intestinimonas*, and *Paludibacter* decreased linearly (*q* ≤ 0.02; Table 4).

**Table 3:** Effect of experimental diets on diversity in phyla in the fecal bacterial community^a^.

**Phylum**	**Treatments**				**SEM^b^**	***P*****-value**		
	**C20**	**C40**	**C60**	**C80**		**Linear**	**Quadratic**	**Cubic**
*Actinobacteria*	0.14	0.26	0.08	0.64	0.145	0.20	0.33	0.30
*Bacteroidetes*	46.59	40.43	39.30	40.35	1.919	0.02	0.06	0.73
Candidatus *Saccharibacteria*	0.04	0.18	0.14	0.02	0.045	0.65	0.01	0.60
*Elusimicrobia*	0.02	0.15	0.14	0.05	0.036	0.80	0.12	0.83
*Fibrobacteres*	0.13	0.14	0.13	0.08	0.016	0.39	0.57	0.92
*Firmicutes*	46.18	45.40	46.33	43.46	0.763	0.29	0.49	0.42
*Proteobacteria*	1.10	0.94	1.61	2.98	0.536	0.02	0.17	0.96
*Spirochaetes*	1.74	4.57	4.08	2.49	0.767	0.71	0.05	0.67
*Verrucomicrobia*	0.92	4.31	5.04	5.80	1.243	0.08	0.49	0.75

a *Only top 10 phyla were displayed, according to the percentage of all sequence data*.

b *SEM, standard error of the mean*.

*C20, diet contained 20% of concentrate; C40, diet contained 40% of concentrate; C60, diet contained 60% of concentrate; C80, diet contained 80% of concentrate*.

**Table 4:** Effect of experimental diets on diversity in genera in the fecal bacterial community^a^.

**Genera**	**Treatments**				**SEM^b^**	***q*****-value**		
	**C20**	**C40**	**C60**	**C80**		**Linear**	**Quadratic**	**Cubic**
*Akkermansia*	0.76	3.69	4.47	5.50	1.175	0.23	0.83	0.99
*Alistipes*	6.64	7.77	6.16	5.61	0.531	0.47	0.78	0.99
*Alloprevotella*	1.34	0.67	1.60	0.15	0.380	0.48	0.83	0.99
*Bacteroides*	7.08	5.92	5.36	5.66	0.434	0.23	0.67	1.00
*Clostridium* IV	1.59	1.41	0.99	2.07	0.257	0.38	0.10	0.83
*Clostridium sensu strict*	0.07	0.07	0.15	0.14	0.012	0.04	0.83	0.83
*Clostridium* XlVa	2.03	2.08	3.62	2.54	0.425	0.31	0.67	0.83
*Clostridium* XlVb	0.79	0.72	0.64	1.18	0.138	0.19	0.27	0.99
*Flavonifractor*	0.64	0.34	0.35	0.45	0.080	0.38	0.49	0.99
*Intestinimonas*	0.74	0.45	0.31	0.10	0.155	< 0.01	0.83	0.99
*Oscillibacter*	1.48	1.98	2.18	2.73	0.298	0.08	0.95	0.99
*Paludibacter*	0.45	0.17	0.07	0.05	0.051	0.02	0.60	0.99
*Paraprevotella*	6.17	2.15	4.03	6.19	1.122	0.85	0.64	0.99
*Phascolarctobacterium*	0.77	1.30	1.52	1.20	0.182	0.47	0.67	1.00
*Prevotella*	1.04	1.01	1.29	0.53	0.184	0.48	0.67	0.99
*Roseburia*	0.15	0.18	0.84	0.94	0.242	0.02	0.93	0.99
*Ruminococcus*	0.26	0.23	0.28	0.44	0.054	0.29	0.70	1.00
*Succinivibrio*	0.03	0.09	0.48	0.81	0.211	0.05	0.83	0.99
*Treponema*	1.73	4.57	4.08	2.49	0.767	0.80	0.49	0.99
Unclassified Ruminococcaceae	24.34	23.74	23.04	19.02	0.644	0.02	0.60	0.99
Unclassified Porphyromonadaceae	5.59	5.16	4.43	3.05	0.400	0.14	0.83	1.00
Unclassified Lachnospiraceae	3.29	3.71	3.95	4.04	0.261	0.48	0.90	1.00
Unclassified Verrucomicrobiaceae	0.15	0.61	0.56	0.28	0.070	0.66	0.18	0.99
Unclassified Prevotellaceae	0.71	0.28	0.41	0.07	0.102	0.21	0.93	0.99

a *Only the genera which were significant different among treatments or the relative abundance of a quarter samples >0.5% were displayed, according to the percentage of all sequence data*.

b *SEM, standard error of the mean*.

*C20, diet contained 20% of concentrate; C40, diet contained 40% of concentrate; C60, diet contained 60% of concentrate; C80, diet contained 80% of concentrate*.

### Sequence and general archaeal community compositions

Following denoising and chimera checking, a total of 829,456 clean sequence reads were generated from 23 fecal samples. The sequence reads, which had an average length of 388 bp among the samples, were assigned to 1,178 OTUs. We obtained an average of 36,063 sequences per animal across the fecal samples, and each sample had an average of 399 OTUs. After normalization to 30,000 sequences, individual sample OTUs ranged from 286 to 523, and 521 OTUs were presented in all the samples (Figure S1B).

Two distinct phyla were were identified in all the fecal samples. The most abundant phylum was Euryarchaeota, which comprised 87.63% of the sequence reads, with only several sequences belonging to Thaumarchaeota as the remaining phylum. Unclassified sequences comprised 12.37% of the total reads. At the genus level, we detected six distinct genera among the treatments, and three genera were common in all samples; these were identified as the core microbiome (Figure S2B). The most abundant known genera were *Methanocorpusculum* (48.17%) and *Methanobrevibacter* (37.67%; Table 5). Other minor genera such as *Methanosphaera, Methanimicrococcus, Methanosarcina*, and *Nitrososphaera* accounted for 0.20, 0.01, < 0.01, and < 0.01%, respectively. The remainder of the sequences (12.37%) were unclassified at the genus level.

**Table 5:** Effect of experimental diets on diversity in genera in the fecal archaeal community.

**Genera**	**Treatments**				**SEM^a^**	***P*****-value**		
	**C20**	**C40**	**C60**	**C80**		**Linear**	**Quadratic**	**Cubic**
*Methanobrevibacter*	49.57	30.07	45.45	26.90	6.459	0.24	0.96	0.14
*Methanosphaera*	0.32	0.23	0.17	0.07	0.061	0.01	0.95	0.76
*Methanocorpusculum*	32.90	57.99	38.14	61.99	8.303	0.19	0.96	0.10
*Methanimicrococcus*	< 0.01	< 0.01	< 0.01	0.03	0.008	0.29	0.66	0.28
*Methanosarcina*	< 0.01	< 0.01	< 0.01	< 0.01	< 0.01	0.21	0.35	0.68
*Nitrososphaera*	< 0.01	< 0.01	< 0.01	< 0.01	< 0.01	0.60	0.26	0.14

a *SEM, standard error of the mean*.

*C20, diet contained 20% of concentrate; C40, diet contained 40% of concentrate; C60, diet contained 60% of concentrate; C80, diet contained 80% of concentrate*.

### Effects of increasing dietary concentrate levels on fecal archaeal communities

Analyses of archaeal 16S rRNA gene fragments revealed no significant differences in richness (Chao1 and PD whole tree) and diversity (Shannon and Simpson indices) with increasing dietary concentrate levels (Table 2). PCoA analysis of overall diversity based on unweighted UniFrac metrics was performed to compare the four treatments (Figure 2B). ANOSIM showed significant difference in archaeal community composition between treatments C80 and C20 (*R* = 0.572, *P* = 0.02). However, there were no significant differences between other treatment comparisons (*P* > 0.10). At the genus level, only the relative abundance of *Methanosphaera* decreased linearly (*P* = 0.01) with increasing dietary concentrate levels (Table 5).

### Changes in the density of fecal microbiota

Quantitative PCR data revealed the relationships between the different levels of dietary concentrate and the populations of microbial taxa (Table 6). However, no significant differences were found in the density of detected fecal microbiome taxa.

**Table 6:** Effect of experimental diets on the fecal microbiota numbers of the heifers.

**Taxa**	**Treatments^a^**				**SEM^b^**	***P*****-value**		
	**C20**	**C40**	**C60**	**C80**		**Linear**	**Quadratic**	**Cubic**
Total bacteria	12.28	12.38	12.34	12.35	0.054	0.69	0.57	0.88
Total Methanogens	7.76	7.42	7.44	7.33	0.093	0.18	0.76	0.33
General *Prevotella*	10.85	10.81	10.82	10.79	0.039	0.70	1.00	0.68
*Fibrobacter succinogenes*	5.50	5.76	5.90	5.80	0.171	0.46	0.62	0.89
*Ruminococcus albus*	5.53	5.70	5.49	5.41	0.256	0.87	0.44	0.84
*Ruminococcus flavefaciens*	9.97	10.04	10.11	10.02	0.113	0.60	0.37	0.65

a *The number of microbes was shown by the log_10_ transfer of the values for gene copies per gram wet weight*.

b *SEM, standard error of the mean*.

*C20, diet contained 20% of concentrate; C40, diet contained 40% of concentrate; C60, diet contained 60% of concentrate; C80, diet contained 80% of concentrate*.

### Correlations between fecal core OTUs and main nutrient and VFAs content

**Figure 3:**
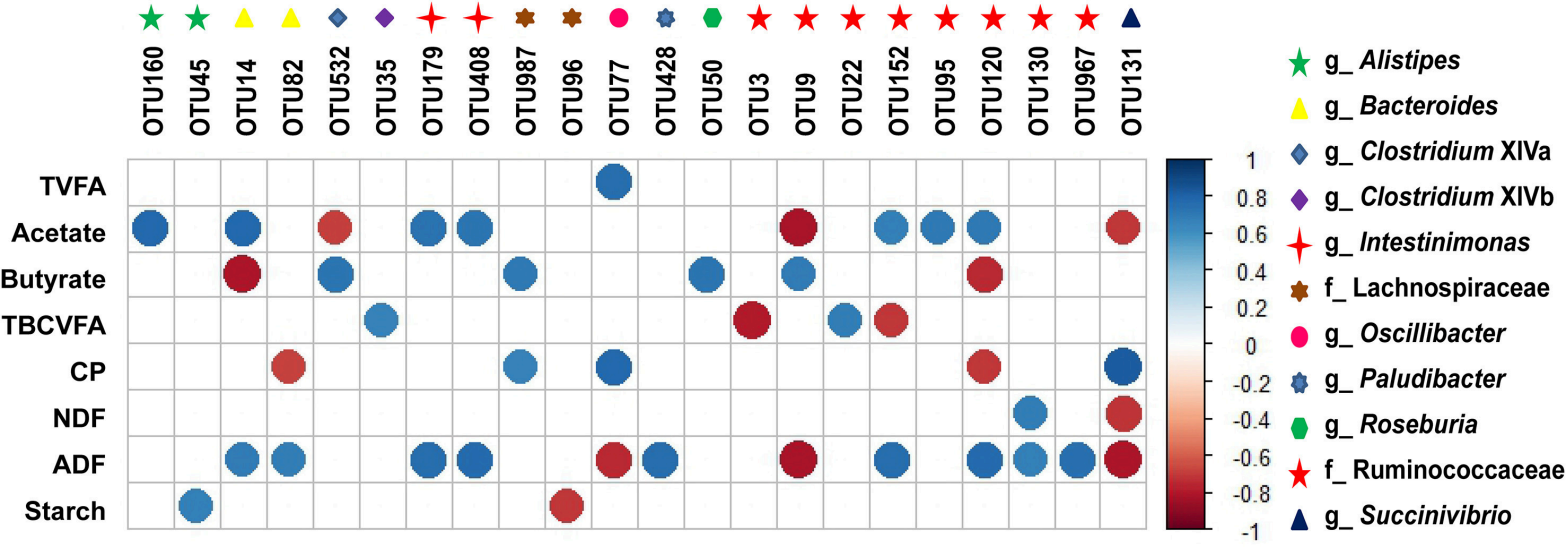
Correlations between fecal core microbiota OTUs and nutrient and VFA content. Spearman non-parametric rank correlation matrix of the core microbiota OTUs and nutrient contents and VFA proportions (molar%). Only significant correlations were shown (*r* > 0.50 or *r* < −0.50 and *q*-value < 0.05). The scale colors denote whether the correlation is positive (closer to 1, dark blue) or negative (closer to −1, dark red) between the OTUs and nutrient and VFA contents. CP, crude protein; NDF, neutral detergent fiber; ADF, acid detergent fiber; TVFA, total volatile fatty acid; TBCVFA, total branch-chain volatile fatty acids; OTUs, operational taxonomic units.

Only one core OTU derived from *Oscillibacter* was positively associated (*r* = 0.76, *q* = 0.01) with total VFA (TVFA) content (Figure 3). Seven core OTUs derived from *Alistipes, Bacteroides, Intestinimonas*, and unclassified Ruminococcaceae were positively (*r* > 0.67, *q* < 0.05) and three core OTUs derived from *Clostridium* XlVa, *Succinivibrio*, and unclassified Ruminococcaceae were negatively (*r* < −0.68, *q* < 0.04) associated with acetate content. Four core OTUs derived from *Clostridium* XlVa, unclassified Lachnospiraceae, *Roseburia*, and unclassified Ruminococcaceae were positively (*r* > 0.69, *q* < 0.04) and two core OTUs derived from *Bacteroides* and unclassified Ruminococcaceae were negatively (*r* < −0.74, *q* < 0.02) associated with butyrate content. Two core OTUs derived from *Clostridium* XlVb and unclassified Ruminococcaceae were positively (*r* > 0.66, *q* < 0.04) and two core OTUs derived from unclassified Ruminococcaceae were negatively (*r* < −0.70, *q* < 0.03) associated with TBCVFA content. Two core OTUs derived from unclassified Lachnospiraceae and *Oscillibacter* were positively (*r* > 0.66, *q* < 0.05) and two core OTUs derived from *Bacteroides* and unclassified Ruminococcaceae were negatively (*r* < −0.67, *q* < 0.04) associated with CP content. One core OTU derived from unclassified Ruminococcaceae was positively (*r* = 0.70, *q* = 0.03) and one core OTU derived from *Succinivibrio* was negatively (*r* = −0.71, *q* = 0.02) associated with NDF content. Nine core OTUs derived from *Bacteroides, Intestinimonas, Paludibacter*, and unclassified Ruminococcaceae were positively (*r* > 0.67, *q* < 0.05) and three core OTUs derived from *Oscillibacter*, unclassified Ruminococcaceae, and *Succinivibrio* were negatively associated (r < −0.74, *q* < 0.02) with ADF content. One core OTU derived from *Alistipes* was positively (*r* = 0.68, *q* = 0.01) and one core OTU derived from unclassified Lachnospiraceae was negatively (*r* = −0.71, *q* = 0.02) associated with starch content.

## Discussion

### Fecal nutrient and VFAs content

As described in our previous study, the intakes of DM, OM, NDF, and ADF decreased linearly with increasing diet concentrate levels, while intakes of starch increased linearly (Zhang et al., 2018). In addition, feed efficiency (average daily gain/dry matter intake) was linearly increased (*P* < 0.01) with increasing dietary concentrate levels (Zhang et al., 2018). These findings might partly explain the changed nutrient content in the feces of the heifers in this study. Because a limit-feeding strategy was used in our study, the TVFA concentrations were a little lower than that in previous reports (Li et al., 2012; Mao et al., 2012, 2015). Similar to the ruminal TVFA concentrations (Zhang et al., 2017), the proportion of fecal acetate decreased in high concentrate diets, while the proportions of fecal propionate and butyrate increased. The butyrate concentration in our study was lower than 8 mM, which is considered as a threshold for inducing severe intestinal epithelial cell apoptosis and disruption of the intestinal barrier (Peng et al., 2007). Moreover, the multiple sampling time points used in our study might have reduced sampling bias as the total feces output (5.33–11.01 kg/d, wet weight) was much greater than the amount of each sample (Mao et al., 2012; Suarez-Mena et al., 2015; Lascano et al., 2016).

### Fecal bacterial community composition

With increasing dietary concentrate levels, fecal bacterial diversity linearly decreased, which agreed with other reports where high proportions of cereals were fed to cattle (Plaizier et al., 2016, 2017). This finding might be due to the lower amounts of available nutrients in the hindgut as concentrates are preferentially fermented in the foregut (Shanks et al., 2011; Li et al., 2012). Our findings showed that *Firmicutes* and *Bacteroidetes* were the most predominant bacteria in feces, which agreed with previous studies (Rice et al., 2012; Li et al., 2017; Plaizier et al., 2017). In addition, the relative abundance of *Firmicutes* and *Bacteroidetes* was comparable to that of the rumen, cecum, and feces in similar studies, where members of the families *Clostridiaceae, Lachnospiraceae, Ruminococcaceae*, and *Prevotellaceae* dominated (de Oliveira et al., 2013; Hua et al., 2017; Plaizier et al., 2017). Previous studies report that the ratios of *Firmicutes* to *Bacteroidetes* are related to energy harvesting and body fat in humans and mice (Ley et al., 2006; Turnbaugh et al., 2006). Meanwhile, in ruminants, fecal *Firmicutes* to *Bacteroidetes* ratios are hardly affected by diets as most of the energy is produced in the foregut rather than the hindgut (Plaizier et al., 2016; Li et al., 2017). Compared with *Firmicutes, Bacteroidetes* has more glycoside hydrolases (GHs) and polysaccharide lyases (PLs) genes per genome and acts as one of the main degraders of many complex polysaccharides in plant cell walls (Meale et al., 2016). In heifers fed high concentrate diets, the competitiveness of *Bacteroidetes* declines allowing other phyla such as Candidatus *Saccharibacteria* and *Proteobacteria* to proliferate faster, resulting in an increase of these two phyla, which may be a reflection of the lower amounts of available fibrous material in the hindgut.

Similarly, unclassified Ruminococcaceae and Lachnospiraceae, *Bacteroides, Alistipes, Akkermansia, Clostridium, Ruminococcus*, and *Prevotella* have been found to predominate in the feces (Callaway et al., 2010; Deng et al., 2017; Li et al., 2017). Unclassified Ruminococcaceae and Lachnospiraceae, *Prevotella*, and *Ruminococcus* have also been detected in 742 rumen samples of 32 types of ruminants from all over the world and might be considered as the “core bacterial microbiome” at the genus level (Henderson et al., 2015). These results indicate that the rumen and feces share a “core bacterial microbiome” at family and/or genus levels and that these bacteria play an important role in the GIT ecosystem (Callaway et al., 2010). However, because the type and amounts of nutrients available to the microbes in the lower tract differ substantially from those in the rumen, it is likely that there are substantial differences between the rumen and the lower GIT with respect to the identity and abundance of individual species within each of these core families and genera (Mao et al., 2015; Popova et al., 2017; Ji et al., 2018). Unclassified Ruminococcaceae and *Ruminococcus*, in the same bacterial family of Ruminococcaceae, both might have important roles in fiber digestion (Russell and Rychlik, 2001; Liu et al., 2016; Shen et al., 2017). Considering the roles of unclassified Ruminococcaceae on fiber digestion (Dowd et al., 2008; Shen et al., 2017), their reduced population in the feces in our study might be due to the reduced fiber content in the hindgut of heifers fed increasing amounts of concentrates. Previous studies also show that the abundance of *Paludibacter* is positively related with dietary forage levels, indicating that they might have the ability to degrade fiber (Nathani et al., 2015; Plaizier et al., 2016). Similarly, our study found a decreased abundance of *Paludibacter* in heifers fed high concentrate diets. These results can be further confirmed by the correlations between most core OTUs derived from unclassified Ruminococcaceae and *Paludibacter* and the fecal NDF and/or ADF contents (Figure 3).

*Bacteroides* are well-known intestinal bacteria with the ability to utilize polysaccharides; they occupy about 5.07–13.7% of cattle feces (Dowd et al., 2008; Callaway et al., 2010; Rice et al., 2012). Previous reports state that *Bacteroides* populations decrease in the feces of cattle fed forage enriched diets (Kim et al., 2014; Nathani et al., 2015; Meale et al., 2016). However, no significant differences were found in the abundance of *Bacteroides* and some other fiber utilizers among the treatments in our study; this might be due to insufficient fiber intake in the high dietary forage groups due to the use of the limit-feeding strategy. *Alistipes* is another common GIT bacteria that was previously classified as a member of the genus *Bacteroides* (Dowd et al., 2008; Kim et al., 2014; Klein-Jöbstl et al., 2014). Therefore, it was reasonable to infer that *Alistipes* might have a similar function to *Bacteroides* in GIT.

*Akkermansia* is reported as one of the important bacteria in the lower intestinal tract with a variety of functions related to glucose homeostasis, mucosa health, lipid and energy metabolism, and inflammation markers; it can also use intestinal mucins to produce acetate and propionate for host intestine epithelial cells (Shin et al., 2014; Schneeberger et al., 2015; Guo et al., 2017). In accordance with other studies, *Akkermansia* predominated in the heifers' feces (Dowd et al., 2008; Li et al., 2017), which might be beneficial to their GIT health, although further investigation is needed.

As a ubiquitous genus in the gastrointestinal tract, *Clostridium* spp. can have both positive and negative health effects on host animals, with the specific effects being related to the individual *Clostridium* species involved (Kopecný et al., 1996; Ozutsumi et al., 2005; Dowd et al., 2008). Even though some members of *Clostridium sensu stricto* are generally perceived as pathogenic, the density of some clostridia can be found up to 10^7^-10^11^ cells/g of intestinal content of healthy individuals (Rajilić-Stojanović and de Vos, 2014). The reasons for the increased abundance of *Clostridium sensu stricto* in the high concentrate diet heifers in our study needs further investigation, and the specific species also requires identification. *Prevotella* produces a variety of extracellular degradative enzymes and is believed to be a starch degrader (Danielsson et al., 2017). In previous studies, cattle fecal *Prevotella* is commonly found associated with dietary, age, and/or seasonal differences (Durso et al., 2012; Kim et al., 2014; Meale et al., 2016). While in this study, the relative abundance of *Prevotella* was only slightly decreased in the feces of the C80 group compared with other groups, which might be due to the higher levels of starch degradation in the foregut.

*Intestinimonas* is a newly described bacterial genus with representative strains present in the GIT of humans, mice, and other animals; they have the ability to convert lysine and sugar into butyrate and acetate (Bui et al., 2016; Durand et al., 2017). *In vitro* tests note that some strains of *Intestinimonas*, when isolated from murine and human intestines, grow poorly in hexose sugars; however, growth is much improved by the addition of acetate (Bui et al., 2016). These findings were confirmed by our study which showed that *Intestinimonas* was significantly positively associated with fecal acetate content (Figure 3). Nevertheless, the relative abundance of *Intestinimonas* decreased linearly with increasing amounts of dietary concentrates, even though high concentrate fed heifers had higher fecal protein and starch contents (Table 1). There might be two reasons for these findings. Firstly, the CP and starch reaching the hindgut might be less available as most of it is insoluble or indigestible (Lukas et al., 2005; Wang et al., 2009). Secondly, the species of *Intestinimonas* in the GIT of ruminants might be different from that in the GIT of humans and mice. Hence, further studies are needed to examine the species level of gut microbiota. To our knowledge, we suggest that this is one of the first studies to report *Intestinimonas* in ruminant GIT.

*Succinivibrio* and *Roseburia* seem to be increased by diets rich in grain, and might contribute to the fermentation of a variety of nonstructural carbohydrates (Kim et al., 2014; Henderson et al., 2015; Plaizier et al., 2017). In accordance, our study found that the abundance of *Succinivibrio* and *Roseburia* increased linearly in the feces of heifers fed increasing levels of dietary concentrates. *Roseburia* was also considered as a butyrate producer (Pryde et al., 2002). These results might partly explain the positive association between *Roseburia* and fecal butyrate content, and the negative association between *Succinivibrio* and fecal NDF and ADF contents (Figure 3). Meanwhile, caution is suggested, because the correlation analyses in this study were based on combined calculations; some non-significant correlations do not indicate there is no real interaction between microbial taxa and nutrient and VFA content, and significant correlations should be confirmed by further experimentation (Berry and Widder, 2014; Patra and Yu, 2015; Shen et al., 2017).

### Fecal archaeal community composition

Euryarchaeota is recognized as the most abundant archaeal phylum in ruminants (Kumar et al., 2015; Danielsson et al., 2017; Zhou et al., 2017). *Methanobrevibacter* is reported to be the most abundant archaeal genus in the rumen as well as in most samples from dairy feces (Kumar et al., 2015; Holman et al., 2016). However, our study found that both *Methanobrevibacter* and *Methanocorpusculum* were predominant in the feces of Holstein dairy heifers. Our results agree with those of Daquiado et al. (2014), who report that *Methanocorpusculum* populations can be found at up to 54.6% in the rectal dung of Hanwoo (*Bos taurus coreanae*). In addition, Jin et al. (2017) found fecal *Methanocorpusculum* could be increased up to 39.4% when active dried yeast is added into diets. These inconsistencies might be due to different animal species, sampling methods, and/or primers used by different authors (Jin et al., 2017). *Methanosphaera* is reported to uniquely generate CH_4_ by reducing methanol with H_2_, and is present at lower levels in the rumen of efficient cattle (Zhou et al., 2009; Jin et al., 2017). Similarly, in our study, *Methanosphaera* abundance decreased linearly in the feces of heifers fed a high concentrate ration and which had been identified as efficient heifers in our previous study (Zhang et al., 2018). Furthermore, the decreased abundance of *Methanosphaera* in feces might be helpful in reducing CH_4_ emission in ruminants (Knapp et al., 2014). Even though the total level of methanogens was similar among treatments, total CH_4_ emission could decrease with the linear decrease in fecal output found with increasing levels of dietary concentrates (Table 1). In a sense, limit-feeding of high concentrate diets could be an efficient and environmentally friendly strategy for heifer rearing (Zanton and Heinrichs, 2009; Zhang et al., 2018).

In summary, this study determined the fecal fermentation parameters, bacterial, and archaeal compositions of heifers limit-fed a wide range of F:C diets based on 16S rRNA gene sequencing. This study demonstrated that different F:C diets had significant effects on the fecal bacterial communities, while they had only small effects on the fecal archaeal communities. The most affected bacterial phyla were *Bacteroidetes* and *Proteobacteria*. The change in core fecal microbiota taxa, such as unclassified Ruminococcaceae, *Intestinimonas, Succinivibrio*, and *Roseburia*, had a close relation with fecal VFAs, NDF, and/or ADF content. These conclusions provide a framework for understanding the microbiota present in the GIT of dairy heifers fed a wide range of dietary F:C ratio, which could be helpful in providing integrative information on their potential for manipulation and relieve some environment problems. Meanwhile, the most beneficial way to implement a limit-feeding strategy with the aim of changing GIT microbiota and subsequently improving feed digestion and/or conversion efficiency still needs further investigation.

## Author contributions

SL, YW, ZC, HY, HS, and JZ conceived and designed the experiments. JZ and HS conducted the experiments and performed all statistical analyses. Finally, the paper was written by JZ, and was revised by SL, YW, ZC, and HY. All authors read and approved the final manuscript.

### Conflict of interest statement

The authors declare that the research was conducted in the absence of any commercial or financial relationships that could be construed as a potential conflict of interest.

## Supplementary material

Table S1 Ingredient and chemical composition of experimental diets. ^1^OM, organic matter; CP, crude protein; NDF, neutral detergent fiber; NFC, non-fibrous carbohydrates; ME, metabolizable energy. ^2^Pre-experimental diet. ^3^Contained 18.50% Ca, 6.00% P, 4.2% Mg, 1.4% K, 2.6% S, 7.5% Na, 12.0% Cl, 30 mg/kg of Se, 0.25% Zn, 0.25% Fe, 0.25% Mn, 1,100 mg/kg of Cu, 15 mg/kg of I, 265,000 IU/kg of vitamin A, 110,200 IU/kg of vitamin D, and 2,300 IU/kg of vitamin E. ^4^NFC = 100 – (NDF + CP + ether extract + ash). ^5^Estimated as ME = total digestible nutrients × 0.04409 × 0.82.

Table S2 Real-time PCR primers used in this study. ^1^W = A or T; B = T or G or C, R = A or G.

Table S3 The identified core OTUs in this study. The numbers of fecal bacterial and archaeal OTUs that appeared in all the samples with an abundance > 0.001 were displayed. OTUs, operational taxonomic units. C20, diet containing 20% of concentrate; C40, diet containing 40% of concentrate; C60, diet containing 60% of concentrate; C80, diet containing 80% of concentrate.

Figure S1 Venn diagram illustrating the overlap of microbial OTUs at 3% dissimilarity level among treatments. Venn diagram of bacteria **(A)** and archaea **(B)** operational taxonomic units (OTUs). C20, diet containing 20% of concentrate; C40, diet containing 40% of concentrate; C60, diet containing 60% of concentrate; C80, diet containing 80% of concentrate.

Figure S2 The shared bacterial and archaeal genera across fecal samples of heifers. **(A)** Relative abundance of shared bacterial genera across all fecal samples. **(B)** Relative abundance of shared archaeal genera across all fecal samples. Percentage is shown on the X-axis. Boxes represent the interquartile range (IQR) between the first and third quartiles (25th and 75th percentiles, respectively); the line inside the box defines the median. Whiskers represent the lowest and highest values within 1.5 times the IQR from the 1st and 3rd quartiles, respectively. Samples with a relative abundance for a given genus exceeding those values are represented as points beside the boxes.
