# Supplementary material for: Effect of Limit-Fed Diets With Different Forage to Concentrate Ratios on Fecal Bacterial and Archaeal Community Composition in Holstein Heifers
Source: Front Microbiol. 2018 May 15;9:976. doi: 10.3389/fmicb.2018.00976 (PMC5962747; doi:10.3389/fmicb.2018.00976)
Supplement: Table S1 — Ingredient and chemical composition of experimental diets. 1OM, organic matter; CP, crude protein; NDF, neutral detergent fiber; NFC, non-fibrous carbohydrates; ME, metabolizable energy. 2Pre-experimental diet. 3Contained 18.50% Ca, 6.00% P, 4.2% Mg, 1.4% K, 2.6% S, 7.5% Na, 12.0% Cl, 30 mg/kg of Se, 0.25% Zn, 0.25% Fe, 0.25% Mn, 1,100 mg/kg of Cu, 15 mg/kg of I, 265,000 IU/kg of vitamin A, 110,200 IU/kg of vitamin D, and 2,300 IU/kg of vitamin E. 4NFC = 100 – (NDF + CP + ether extract + ash). 5Estimated as ME = total digestible nutrients × 0.04409 × 0.82. [file Data_Sheet_1.docx]

Supplement Table S1 Ingredients and chemical composition of experimental diets

| Item ^1^ | Dietary concentrate inclusion (%) | | | | |
| --- | --- | --- | --- | --- | --- |
|  | 20 | 40 | 60 | 80 | 50 ^2^ |
| Ingredients, % of DM |  |  |  |  |  |
| Steam-flaked corn | 5.76 | 22.97 | 40.26 | 57.06 | 31.50 |
| Soybean meal | 11.39 | 13.86 | 16.05 | 18.66 | 15.04 |
| Corn silage | 80.00 | 60.00 | 40.00 | 20.00 | 50.00 |
| Mineral mix ^3^ | 2.85 | 3.17 | 3.69 | 4.28 | 3.46 |
| Chemical composition |  |  |  |  |  |
| OM, % of DM | 92.9 | 93.0 | 93.0 | 92.8 | 93.0 |
| CP, % of DM | 12.9 | 13.8 | 14.6 | 15.5 | 14.2 |
| NDF, % of DM | 45.6 | 36.7 | 27.8 | 19.0 | 32.3 |
| NFC ^4^, % of DM | 30.5 | 38.8 | 47.5 | 54.6 | 43.7 |
| Starch, % of DM | 26.3 | 32.3 | 38.2 | 43.8 | 35.1 |
| ME ^5^, Mcal/kg | 2.4 | 2.6 | 2.8 | 2.9 | 2.7 |
| CP:ME (g/Mcal) | 52.9 | 52.9 | 52.7 | 52.7 | 52.8 |

^1^ OM, organic matter; CP, crud protein; NDF, neutral detergent fiber; NFC, non-fiberous carbohydrates; ME, metabolizable energy.

^2^ Pre-experimental diet.

^3^ Contained 18.50% Ca, 6.00% P, 4.2% Mg, 1.4% K, 2.6% S, 7.5% Na, 12.0% Cl, 30 mg/kg of Se, 0.25% Zn, 0.25% Fe, 0.25% Mn, 1,100 mg/kg of Cu, 15 mg/kg of I, 265,000 IU/kg of vitamin A, 110,200 IU/kg of vitamin D, and 2,300IU/kg of vitamin E.

^4^ NFC = 100 – (NDF + CP + ether extract + ash).

^5^ Estimated as ME = total digestible nutrients × 0.04409 × 0.82.

Supplement Table 2 Real-time PCR primers used in this study

| Target | Primer sequence (5’-3’) ^1^ | Product size (bp) | Annealing temperatures (°C) | Annealing time (s) | References |
| --- | --- | --- | --- | --- | --- |
| Total bacteria | F ACTCCTACGGGAGGCAGCAG  R TTACCGCGGCTGCTGGCAC | 195 | 60 | 30 | ([Huse et al., 2008](#_ENREF_2)) |
| Total Methanogens | F TTCGGTGGATCDCARAGRGC  R GBARGTCGWAWCCGTAGAATCC | 140 | 60 | 60 | ([Tan et al., 2011](#_ENREF_4)) |
| General *Prevotella* | F GGTTCTGAGAGGAAGGTCCCC  R TCCTGCACGCTACTTGGCTG | 121 | 55 | 60 | ([Stevenson and Weimer, 2007](#_ENREF_3)) |
| *Fibrobacter succinogenes* | F GTTCGGAATTACTGGGCGTAAA  R CGCCTGCCCCTGAACTATC | 121 | 60 | 34 | ([Denman and McSweeney, 2006](#_ENREF_1)) |
| *Ruminococcus albus* | F CCCTAAAAGCAGTCTTAGTTCG  R CCTCCTTGCGGTTAGAACA | 176 | 60 | 34 | ([Denman and McSweeney, 2006](#_ENREF_1)) |
| *Ruminococcus ﬂavefaciens* | F GATGCCGCGTGGAGGAAGAAG  R CATTTCACCGCTACACCAGGAA | 286 | 60 | 34 | ([Denman and McSweeney, 2006](#_ENREF_1)) |

^1^ W = A or T; B = T or G or C, R = A or G.
