# Supplementary figures and images for: Effect of Limit-Fed Diets With Different Forage to Concentrate Ratios on Fecal Bacterial and Archaeal Community Composition in Holstein Heifers
Source: Front Microbiol. 2018 May 15;9:976. doi: 10.3389/fmicb.2018.00976 (PMC5962747; doi:10.3389/fmicb.2018.00976)

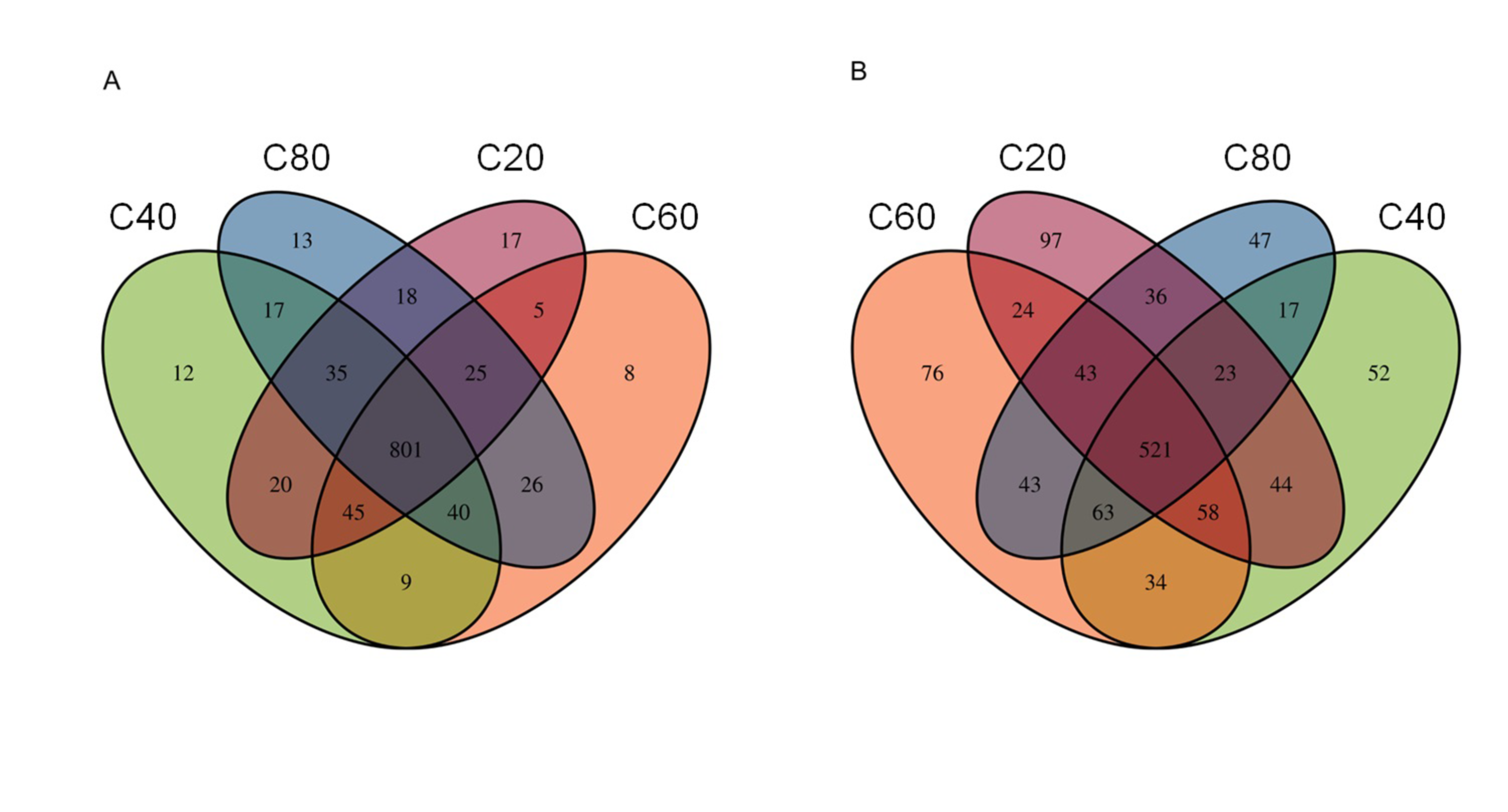

Supplement: Figure S1 — Venn diagram illustrating the overlap of microbial OTUs at 3% dissimilarity level among treatments. Venn diagram of bacteria (A) and archaea (B) operational taxonomic units (OTUs). C20, diet containing 20% of concentrate; C40, diet containing 40% of concentrate; C60, diet containing 60% of concentrate; C80, diet containing 80% of concentrate. [file Image_1.TIF]

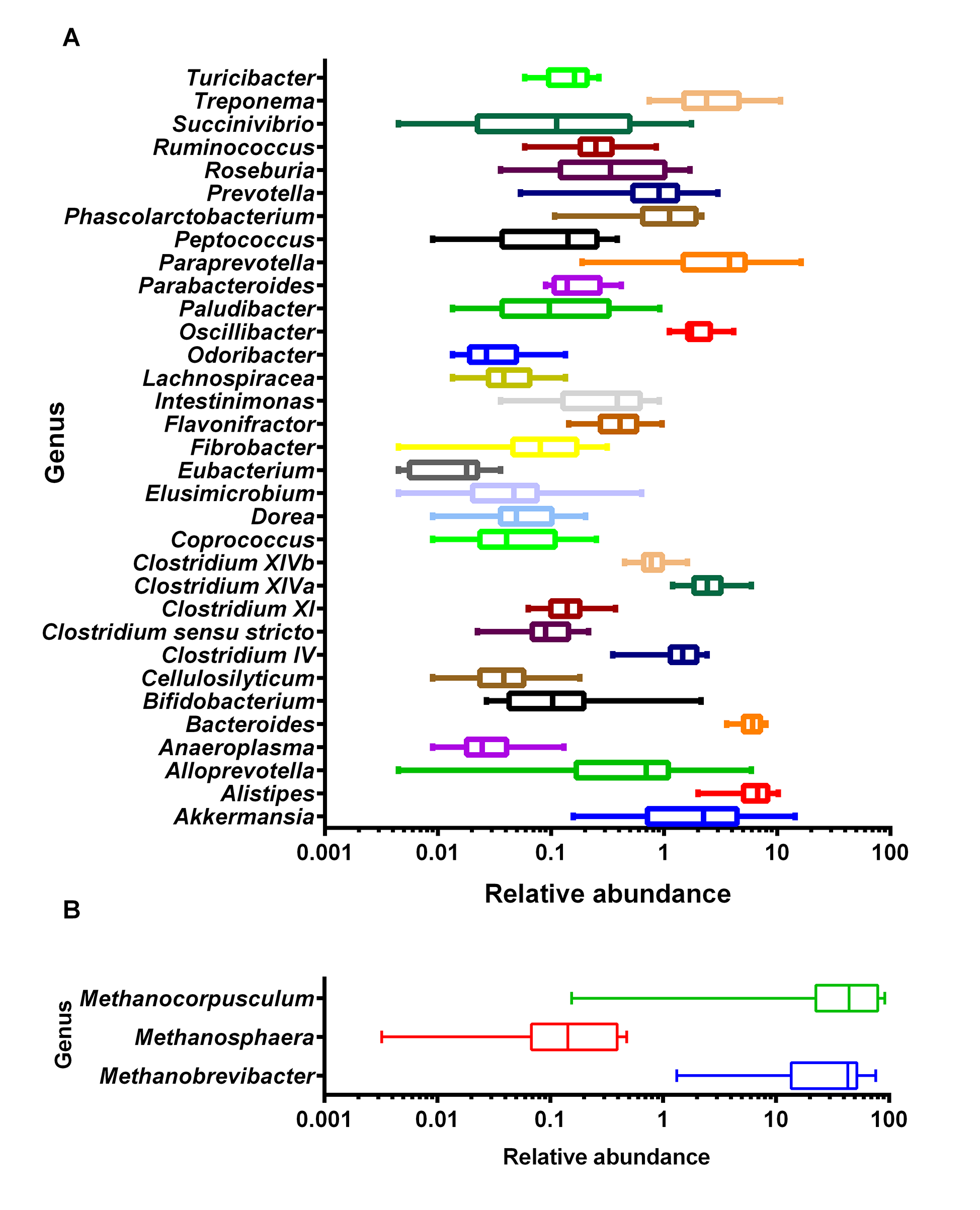

Supplement: Figure S2 — The shared bacterial and archaeal genera across fecal samples of heifers. (A) Relative abundance of shared bacterial genera across all fecal samples. (B) Relative abundance of shared archaeal genera across all fecal samples. Percentage is shown on the X-axis. Boxes represent the interquartile range (IQR) between the first and third quartiles (25th and 75th percentiles, respectively); the line inside the box defines the median. Whiskers represent the lowest and highest values within 1.5 times the IQR from the 1st and 3rd quartiles, respectively. Samples with a relative abundance for a given genus exceeding those values are represented as points beside the boxes. [file Image_2.TIF]
